# Large-Scale Direct Growth of Monolayer MoS_2_ on Patterned Graphene for van der Waals Ultrafast Photoactive Circuits

**DOI:** 10.1021/acsami.4c07028

**Published:** 2024-07-12

**Authors:** Rahul Sharma, Henry Nameirakpam, David Muradas Belinchón, Prince Sharma, Ulrich Noumbe, Daria Belotcerkovtceva, Elin Berggren, Viliam Vretenár, Lubomir Vanco, Matus Matko, Ravi K. Biroju, Soumitra Satapathi, Tomas Edvinsson, Andreas Lindblad, M. Venkata Kamalakar

**Affiliations:** † Department of Physics and Astronomy, Uppsala University, Box 516, Uppsala SE-751 20, Sweden; ‡ Department of Physics, Indian Institute of Technology Roorkee, Roorkee 247667, India; ∥ Centre for Nanodiagnostics of Materials, Faculty of Materials Science and Technology, Slovak University of Technology, Vazovova 5, Bratislava 812 43, Slovakia; ⊥ School of Advanced Sciences−Division of Physics, Vellore Institute of Technology, Vandalur−Kelambakkam Road Chennai, Chennai, Tamil Nadu 600127, India; # Department of Materials Science and Engineering, Uppsala University, Box 35, Uppsala SE-751 03, Sweden; ∇ Université de Strasbourg, CNRS, Institut de Physique et Chimie des Matériaux de Strasbourg (IPCMS), UMR 7504, 23 rue du Loess, Strasbourg 67034, France

**Keywords:** Graphene, TMDs, Field effect transistor, van der Waals heterostructure, ultrafast, photoactive circuits

## Abstract

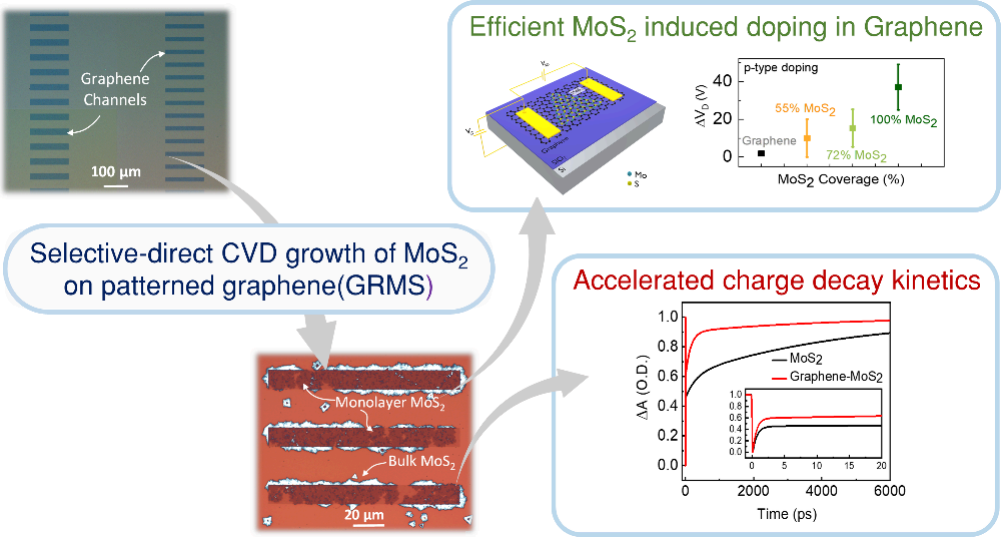

Two-dimensional (2D) van der Waals heterostructures combine the distinct properties of individual 2D materials, resulting in metamaterials, ideal for emergent electronic, optoelectronic, and spintronic phenomena. A significant challenge in harnessing these properties for future hybrid circuits is their large-scale realization and integration into graphene interconnects. In this work, we demonstrate the direct growth of molybdenum disulfide (MoS_2_) crystals on patterned graphene channels. By enhancing control over vapor transport through a confined space chemical vapor deposition growth technique, we achieve the preferential deposition of monolayer MoS_2_ crystals on monolayer graphene. Atomic resolution scanning transmission electron microscopy reveals the high structural integrity of the heterostructures. Through in-depth spectroscopic characterization, we unveil charge transfer in Graphene/MoS_2_, with MoS_2_ introducing p-type doping to graphene, as confirmed by our electrical measurements. Photoconductivity characterization shows that photoactive regions can be locally created in graphene channels covered by MoS_2_ layers. Time-resolved ultrafast transient absorption (TA) spectroscopy reveals accelerated charge decay kinetics in Graphene/MoS_2_ heterostructures compared to standalone MoS_2_ and upconversion for below band gap excitation conditions. Our proof-of-concept results pave the way for the direct growth of van der Waals heterostructure circuits with significant implications for ultrafast photoactive nanoelectronics and optospintronic applications.

## Introduction

Starting from graphene, two-dimensional (2D) materials with diverse electronic properties have evolved as promising candidates for next-generation electronic,^1^ spintronic,^2−4^ and optoelectronic applications.^5^ The discovery and utilization of 2D materials have led to the era of quantum metamaterials, which are not simple or complex compounds but are assembled layer by layer in carefully arranged van der Waals heterostructures^6,7^ that are stacks of 2D materials with contrasting properties.^2,3,8,9^ Moreover, the engineering of homo- and heterostructures with precise interlayer orientations has recently facilitated the emergence of moiré materials for the correlated matter.^10^ In these materials, beyond the dynamics of free electrons or spins, novel correlated quantum states of electrons and spin, such as superconductivity in magic-angle bilayer graphene,^11^ exotic Wigner crystals^12^ (crystals of electrons), 2D magnetism, and novel magnetic textures,^13^ can be realized and controlled electrically for the first time in tunable lattice heterostructures. For standardizing the experimental observations and scalability of quantum and spin transport for practical applications, large-scale 2D material growth is of primordial importance. In this context, significant developments have been performed regarding the growth and testing of the performance of large-scale chemical vapor-deposited materials.^14−17^ Various 2D materials have been successfully grown, including graphene and 2D semiconductors such as MoS_2_, WS_2_, and 2D magnets.^17−20^ Despite these, state-of-the-art reports of heterostructures predominantly rely on wet or dry transfer methods, both of which result in polymer residues at the interfaces that impact the longevity and device performance. The CVD growth of heterostructures remains challenging due to the complex surface physics, extremely small grain size, lack of monolayer growth, and problems achieving efficient interlayer coupling and charge transfer.^21−25^ To ensure the reliability and scalability of such applications, there is a need to establish direct growth methods for 2D material heterolayers with optimal interfaces. The interface between different 2D materials plays a crucial role in various processes such as charge transfer, spin injection, interlayer exciton formation, and proximity-induced effects. Therefore, achieving atomically smooth and defect-free interfaces in heterostructures is paramount for these applications. Various combinations of two-dimensional heterostructures, such as WS_2_/MoS_2_, Graphene/MoS_2_, Graphene/WS_2_, and WS_2_/NbS_2,_ are promising for unique applications in diverse fields like optoelectronics, photovoltaics, and spintronics.^26,27^ Notably, out of all of the heterostructures, Graphene/MoS_2_ (GRMS) heterostructures have shown versatile utility in optoelectronic, spintronic, and optospintronic applications, stemming from the presence of a high spin–orbit coupling in MoS_2_ together with a direct band gap, in sharp contrast to graphene exhibiting complementary properties. For instance, a synergistic combination of the optical properties of MoS_2_ and the chemical stability of fluorographene in a MoS_2_/fluorographene heterostructure has yielded ultrastable and wide-range photodetectors.^28^ While the band gap allows for sharp photoresponse, the spin–orbit coupling in MoS_2_ is key for spin active (in which electron spin can be manipulated) and optospintronic circuits. Thus, by leveraging the long spin diffusion length of graphene and the ability of MoS_2_ monolayers to inject polarized spin carriers from different valleys, GRMS heterostructures have been employed for optospintronic demonstrations.^26,29^ Considering that large-scale CVD graphene shows competitive charge and spin transport properties,^30−32^ this demonstration could be translated into practical applications if direct growth of MoS_2_ on patterned graphene channels is realized, a key challenge we address in this work for the first time.

In this work, we demonstrate a direct growth of van der Waals heterostructures of monolayer graphene and monolayer MoS_2_ (GRMS) employing a confined space chemical vapor deposition technique performed on patterned CVD graphene to enhance control over vapor transport and restrict the growth to a monolayer of MoS_2_. We perform comprehensive structural, spectroscopic, and electrical measurements of graphene and GRMS heterostructures to study the electronic quality of individual layers and how MoS_2_ growth impacts the underlying graphene. These experiments allow us to see the intrinsic doping of MoS_2_ on graphene and interlayer strain that leads to enhanced coupling with preserved electronic quality of the materials. Photoconductivity and time-resolved graphene–MoS_2_ charge transfer dynamics establish the quality of the heterostructures and provide an understanding of the underlying unique charge transfer and upconversion processes. These experiments unveil new insights offering a significant advancement in developing electronic materials and new implications for scalable 2D heterostructure photoactive electronic circuits.

## Results and Discussion

**Figure 1:**
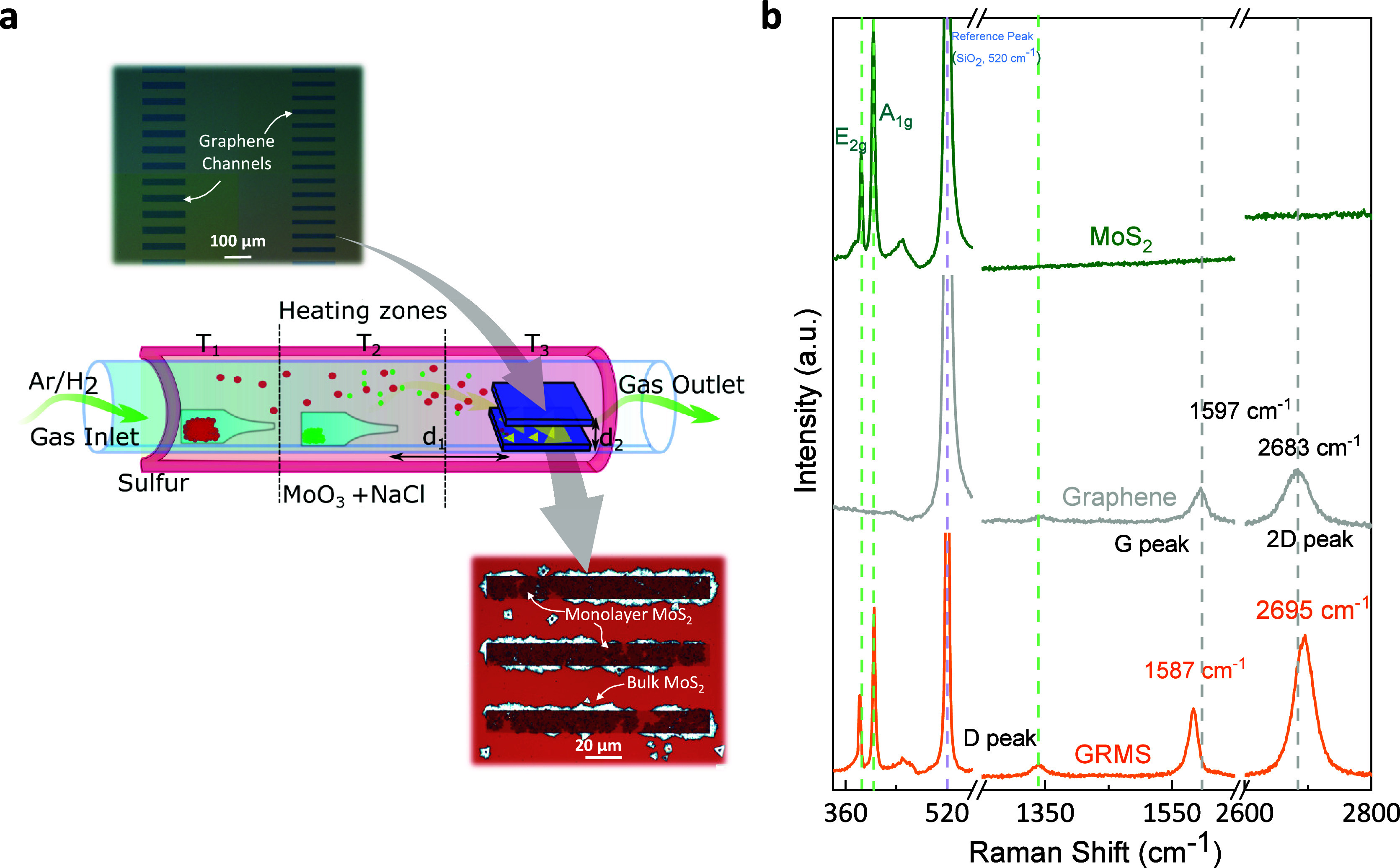
(a) Process scheme depicting patterned graphene, growth of MoS_2_ in the T3 zone, and final grown Graphene/MoS_2_ heterostructure. (b) Comparison of Raman spectra of MoS_2_ (MS, green color), graphene (GR, gray color), and Graphene/MoS_2_ heterostructure (HS, orange).

Graphene/MoS_2_ heterostructures were synthesized using unique confined space chemical vapor deposition for precise control over vapor parameters. Figure 1a illustrates a scheme of the heterostructure growth. To explore direct selective growth of the heterostructures on only graphene channels, first, a monolayer of commercial CVD graphene on Si/SiO_2_ substrate was patterned into arrays of rectangular stripes. Next, the patterned graphene stripes were loaded into a three-zone CVD furnace, where MoS_2_ was synthesized within a confined space of the graphene stripes in an inert atmosphere, utilizing MoO_3_ and sulfur powder as precursors. Various growth parameters, such as *d*_1_ of the confined space (determining the volume of the confined space) and *d*_2_ (the distance between the precursors and the substrate), temperature, and pressure, among others, were optimized to obtain monolayer MoS_2_ growth. Before growth on patterned graphene stripes, we optimized large-area growth monolayer MoS_2_ over extensive graphene (Figure S1a). The final Graphene/MoS_2_ heterostructures grown directly on patterned graphene are displayed in Figure 1a (lower right), illustrating a preferential monolayer growth of MoS_2_ on the patterned CVD graphene stripes. The smooth surface of graphene with fewer dangling bonds, in contrast to the Si/SiO_2_ substrate with less surface energy, facilitates the self-limiting and preferential growth of predominantly monolayer MoS_2_ over stripes of graphene. The size of the MoS_2_ crystals can be reduced by strain induced lattice mismatch between graphene and MoS_2_ as well as thermal strain introduced by the substrate.^33^ Adjacent to the graphene stripes, bulk MoS_2_ growth occurs, which can be attributed to the presence of a step-formed Si/SiO_2_ on the substrate, the reactivation effect of passivated graphene edges due to annealing, and likely enhanced dangling bonds resulting from the etching process of graphene. Figure S1b shows MoS_2_ nearly covering the whole graphene stripes and multilayer growth on the edges of the graphene. This is further illustrated with field emission scanning electron microscopy (FESEM) and detailed atomic force microscopy (AFM) images (Figure S2). AFM images provide clear evidence of multilayer MoS_2_ growth along the grain boundaries, indicating that these locations are primary sites for the commencement of growth. The MoS_2_ crystals merge together through chemical bonding, forming a continuous monolayer over graphene stripes.^34^ For MoS_2_, the primary defects are single or double chalcogen vacancies (sulfur vacancies), which can be minimized through post annealing in an abundant supply of sulfur. The direct growth process is promising for realizing an atomically smooth interface between these two 2D materials, free from residual or polymeric contamination. At the same time, the growth of MoS_2_ on patterned graphene paves the way for custom-grown heterostructure-integrated graphene circuits. The successful formation of the GRMS heterostructure was confirmed through Raman spectroscopy (see Figure 1b), where it was compared to pristine MoS_2_ and graphene samples. The Raman spectra of pristine MoS_2_ exhibited characteristic E_2g_ (in-plane vibration mode) and A_1g_ (out-of-plane) modes at 385 and 404 cm^–1^, respectively, indicating the presence of monolayer MoS_2_.^28,35^ The difference observed in the E_2g_:A_1g_ intensity ratios of GRMS and pristine MoS_2_ (Table S1) can be attributed to the change in doping of MoS_2_ in the heterostructure. Conversely, pristine graphene predominantly exhibited E_2g_ (in-plane vibration of the sp^2^ carbon in graphene, G peak) and a double-resonance 2D peak at 1597 and 2683 cm^–1^, respectively.^36^ Compared to pristine graphene, the enhanced D peak at 1350 cm^–1^ shows enhanced Raman-active sp^3^ bonds in graphene. Such an increase indicates a partial defect creation via sp^3^ bonding with an overlayer of MoS_2_. At the same time, such bonding can also preferably occur with carbon atoms at the grain boundaries and defects where thicker MoS_2_ growth can occur. In this case, the Raman spectra exhibited the signatures of both MoS_2_ and graphene, albeit with slight shifts in both directions. These shifts primarily arise from the monolayers’ strain and charge transfer interactions, analyzed in the latter part of this manuscript. The controlled growth process of the GRMS heterostructures, along with the distinct Raman-active peaks and their preserved relative intensities ratio, underscores its significance for diverse device concepts in 2D circuits.

**Figure 2:**
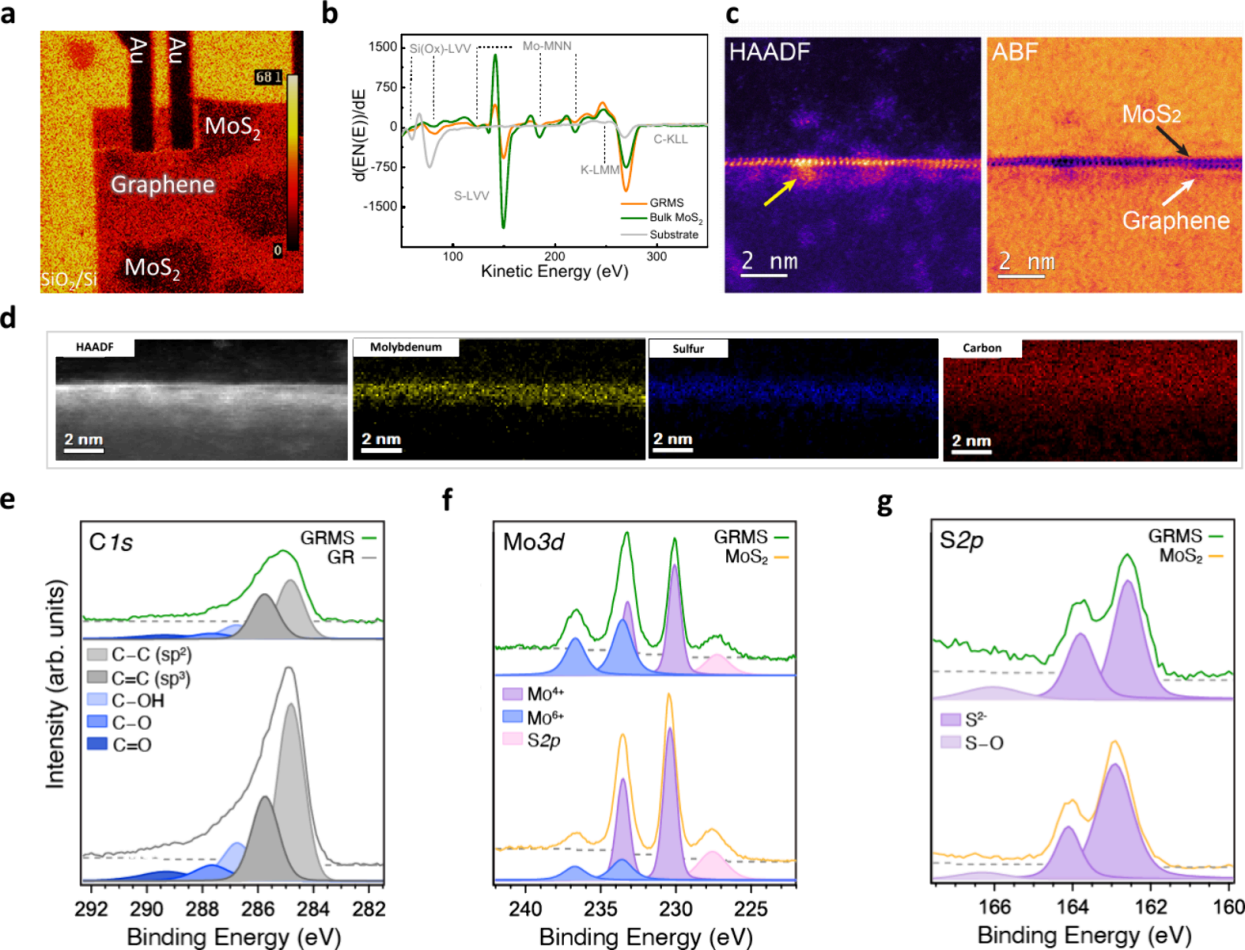
(a) Auger mapping of the GRMS heterostructure illustrates contrast for monolayer graphene, MoS_2_, and gold electrodes. (b) Auger spectra were collected from the GRMS sample, bulk MoS_2_, and Si/SiO_2_ substrate. (c) HAADF and ABF STEM images depict a GRMS heterostructure cross-section prepared on a Si/SiO_2_ substrate. (d) EELS maps of molybdenum, sulfur, and carbon at the vdW interface. XPS spectra and their corresponding fits for core levels of (e) C 1s, (f) Mo 3d, and (g) S 2p.

Our sample was further characterized using Auger spectroscopy (Figure 2a and 2b). Due to its exceptional sensibility for thin layers, Auger spectroscopy is perfectly suited for inspecting the local composition of 2D layered structures. The reduction of the Auger signal, represented by intensity *I*, from a uniform material covered by a thin layer with thickness *t* follows an exponential pattern described by the formula *I* = *I*_0_ exp(−*t*/λ cos θ). Here, *I*_0_ is the intensity of an uncovered material and λ is the attenuation length of electrons at the energy of the specific Auger transition in the corresponding solid.^37^ For practical purposes, parameter λ can be estimated from the TPP-2 M equation. The Auger mapping of the Si oxide LVV transition at ∼70 eV (as shown in Figure 2b) reveals distinct features. Uncovered SiO_2_ areas exhibit a strong signal (indicated by bright yellow in Figure 2a). This observation is attributed to the minimal inelastic mean free path of Auger electrons at a kinetic energy of 70 eV, typically ∼0.5 nm. Consequently, regions covered with graphene display a reduced signal, while areas overlaid with MoS_2_ flakes appear dark. In contrast, the Auger maps for the S-LVV, C-KLL, and Au-MNN transitions (depicted in Figure S3) exhibit heightened signals in regions with MoS_2_, graphene, and gold. The Auger spectra in Figure 2b are acquired from typical positions representing pure SiO_2_, SiO_2_/MoS_2_, and SiO_2_/GRMS. By analyzing the intensity of the Si oxide LVV transition using *I*, we deduce the thicknesses of the deposits to be ∼0.9 nm for the heterostructure layer within the graphene stripe, confirming the extensive monolayer growth of MoS_2_ over graphene stripes and 1.2 nm for the MoS_2_ bilayer on SiO_2_ outside the graphene stripe. Residual potassium traces can be attributed to remnants from salt-assisted growth. Optimal quantity of salt is known to promote an increased supply of Mo precursors and is advantageous for uniform MoS_2_ growth.^38,39^ Furthermore, the epitaxial monolayer growth of MoS_2_ over graphene is corroborated through high-angle annular dark-field scanning transmission electron microscopy (HAADF-STEM) analysis of the GRMS cross-section, as detailed in the Experimental Section.

In Figure 2c, the high-angle annular dark field (HAADF) and annular bright-field (ABF) scanning transmission electron microscopy (STEM) images reveal monolayer MoS_2_, identifiable by the HAADF atomic intensities corresponding to “Mo” and “S” atoms, exhibiting a distinctive trigonal prismatic structure. However, the substrate waviness partially hinders the perfect projection of the monolayer. MoS_2_ and underlying graphene are represented by black and white arrows, respectively. Notably, the intensity of the atomic columns of graphene (low-*Z* carbon atoms) is much lower due to the HAADF intensity power law, *I* ∝ *z*^α^, where α can be equal to 1.5 or 2.^40^ As a result, the graphene layer is imperceptible in HAADF imaging. On the contrary, ABF imaging, being more sensitive to low-*Z* elements, clearly displays the graphene layer, as supported by the corresponding intensity line profile presented in the Supporting Information (Figure S4a and S4b). In addition, marginal contamination is observed very close to the graphene and MoS_2_ layer (shown by a yellow-colored arrow in the HAADF image), likely occurring during the direct CVD growth process in the form of unreacted S and Mo precursors. Further, we have investigated this atomic level of contamination at the interface by electron energy loss spectroscopy (EELS), as shown in Figure S4d. Figure 2d shows the corresponding EELS maps that depict the presence of Mo, S, and C elements at the interface. Quantification of the spectrum has resolved nearly ideal stoichiometry for the MoS_2_ structure (see Table S2 in the Supporting Information). Interestingly, the elemental mapping at the MoS_2_–graphene interface is in good agreement with HAADF-STEM (see Figure 2c). Thus, the dark-field and bright-field images in Figure 2c unmistakably illustrate the atomically smooth growth of a monolayer of MoS_2_ over graphene. Figure S5 shows the top view of the HAADF and BF images of a GRMS and pristine graphene.

We conducted hard X-ray photoelectron spectroscopy to chemically characterize the heterostructure and compare it with pristine MoS_2_ and graphene (Figure S6 compares the entire spectra). In Figure 2d, 2e, and 2f, we present the core-level spectra of C 1s, Mo 3d, and S 2p, respectively, along with their corresponding fitted peak components. Figure 2e contrasts the C 1s spectra of graphene and the heterostructure. Both exhibit a prominent sp^2^ C–C peak at 284.8 eV, confirming the good quality of graphene and no influence from oxides. Additionally, the C 1s spectra show a robust sp^3^ peak at 285.7 eV.^41^ A description on line positions and assigned compounds is presented in Table S3. The GRMS samples show a nearly 10% increase in sp^3^ carbon presence within the graphene compared to the pristine counterpart, suggesting a modest rise in sp^3^ surface defects on the graphene during growth. This increase in sp^3^ carbon contributes to increased binding energy and can induce a more distinctive p-type characteristic within the graphene. Given the X-ray probe’s large size (100 μm), the sp^3^ contribution originating from other sources (graphitization of the surface or resist residues) cannot be ruled out. However, the overall enhancement of sp^3^ can be ascribed to the MoS_2_ growth over the graphene, as confirmed by subsequent micro-Raman analysis discussed in the following section.

**Figure 3:**
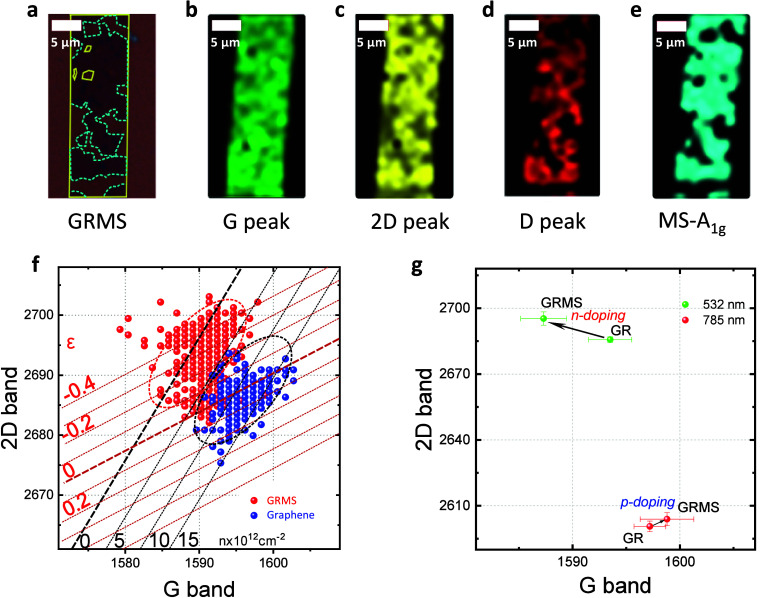
(a) Optical image of Graphene/MoS_2_ (HS). The yellow solid line shows the patterned graphene, and the dotted cyan-colored line represents the growth of monolayer MoS_2_. Raman mapping of HS for the (b) G band, (c) 2D band, and (d) D band of graphene (GR), and (e) A1g mode of MoS_2_ (MS). (f) Correlation plot of the position of the 2D peak as a function of the position of the G peak in GR and HS. Zero-strain and zero-doping lines are taken from ref (42). (g) Wavelength-dependent charge transfer behavior in Graphene/MoS_2_. Note: Measurements in Figure 3g were conducted on a sapphire substrate due to the challenging observation of graphene Raman spectra on silicon substrates with a 785 nm wavelength.

The phenomena of sp^3^ bonding and charge transfer are intrinsic to the vapor deposition of materials with differing work functions onto graphene.^43^ We have investigated the charge transfer across the graphene–MoS_2_ interface by examining the mechanical and electronic interplay within the heterostructure using Raman spectroscopy. Figure 3a presents an optical micrograph of the heterostructure featuring a monolayer of MoS_2_ grown over a graphene layer. For a guide to the eye, the graphene stripe is demarcated by a plain yellow boundary while the MoS_2_ region is indicated by a cyan-colored dashed line. Figure S7 shows an AFM image of MoS_2_ crystals grown on a graphene single domain predominantly aligned in the same direction. Figure 3b–d reveals the characteristic peak intensities in the Raman mappings corresponding to graphene’s G, 2D, and D peaks, respectively, while Figure 3e showcases the A_1g_ peak of MoS_2_. In Figure 3b, the visual uniformity of the graphene within the stripe is evident. Figure 3c and 3d consistently aligns with the growth pattern of MoS_2_. Notably, the sharp intensity contrast observed in Figure 3c (corresponding to the 2D peak) stems from a frequency shift in the peak position of the 2D peak of approximately 12 cm^–1^, as elucidated in Figure 1b. Figure 3b–e collectively presents the confined growth of MoS_2_ on the graphene stripe. Furthermore, a Raman-active defect D peak emerging with MoS_2_ growth could signify a possible covalent interaction between the two 2D monolayers. In the Mo 3d and S 2p XPS spectra, features at the chemical shift that would indicate Mo–C and S–C are absent.^44,45^ The detection limit of XPS is about 1%, which puts an upper bound on the number of such bonds that exist, even though the Raman D peak suggests their presence. The XPS data in Figure 2d and Table S3 suggests the potential enhancement of sp^3^ carbon in the graphene structure beneath the MoS_2_ layer.

**Figure 4:**
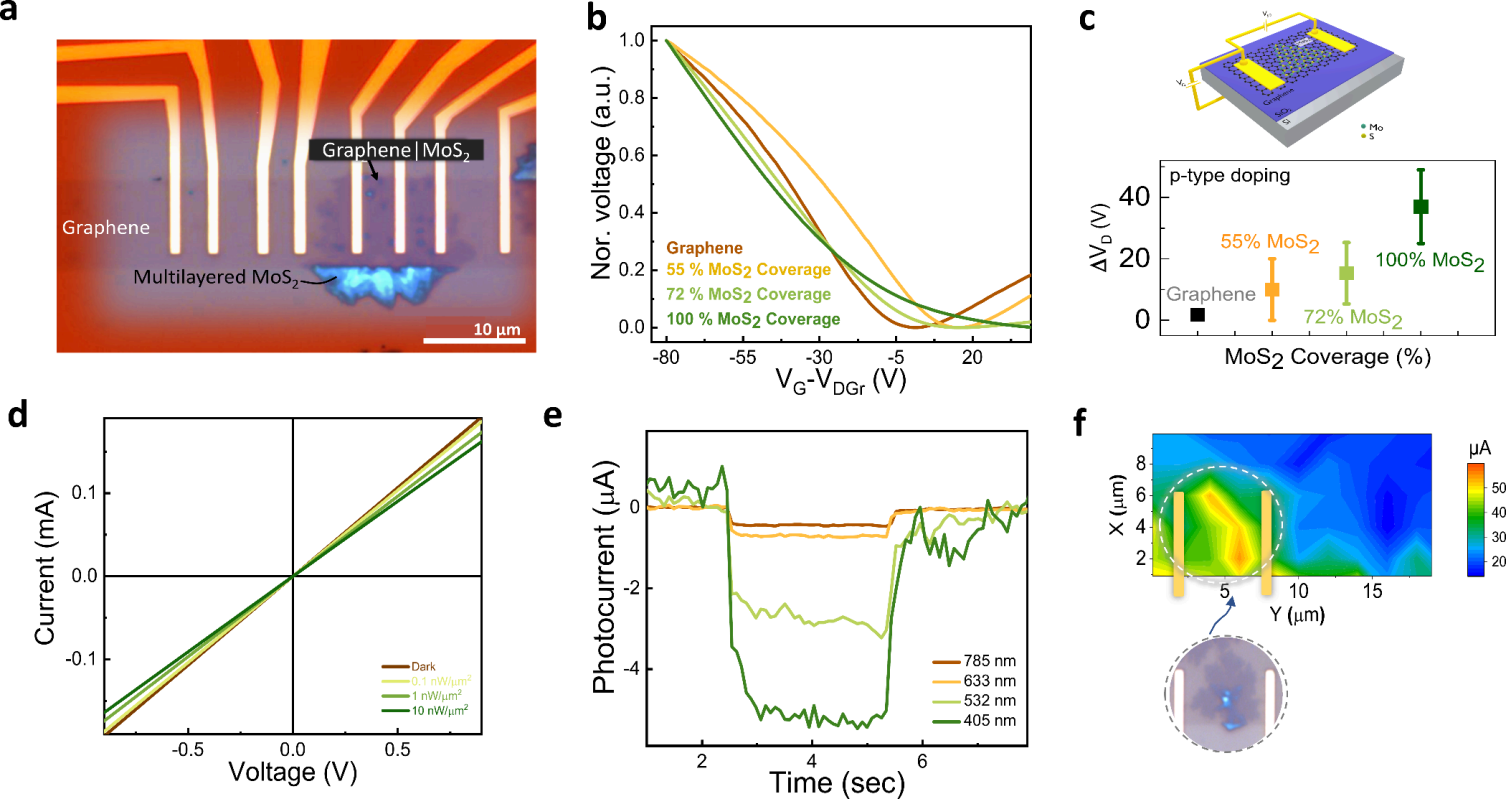
Electrical and photoconductivity measurements. (a) Optical image of a multiterminal GRMS device. (b) Back-gate electrical measurements in a four-probe configuration displaying the normalized drain-to-source voltage as a function of the applied gate voltage. The measurements were taken for both bare graphene and regions covered by 55%, 72%, and 100% monolayer MoS_2_, with gate sweeps conducted at *I*_ds_ = 10 μA. (c) (Top) Schematic illustration of back-gated field effect transistors (FETs) based on GRMS with contacts positioned on the graphene layer and the channel covered by MoS_2_. (Bottom) Dirac point shift with corresponding percentages of MoS_2_ coverage over the graphene channel. (d) Photoresponse of the device shown in Figure 4f at different laser power with a 532 nm laser. (e) Wavelength-dependent time response from the same device showing the spectral response of the device at 0.1 nW/μm^2^. (f) A device with ∼70% MoS_2_ area coverage depicting the photoresponse map.

The charge transfer and strain are quantified by a strain-doping plot for graphene (see Figure 3f). The strain and doping lines are plotted considering the dependence of the Raman frequencies on both strain and doping to a linear relation to some extent. The Raman frequencies (ω) of any solid (G and 2D in the case of graphene) are linearly proportional to the lattice strain (ε) by the Grüneisen parameter (γ) Δω = −2γω_0_ε, where ω_0_ is the frequency at zero strain.^42,46^ The experimental values of γ for G and 2D were found to be 1.9 and 2.6, respectively.^42,47^ Next, the Raman frequencies for graphene are found to be quasilinear with both the electron and the hole, which implies frequencies and carrier concentration as *ω = k*_*n*_*n,* where *n* is the carrier concentration and *k*_*n*_ is a linearity constant. The *k*_*n*_ values are −9.6 × 10^13^ and −1 × 10^13^ for G and 2D respectively. Adding eqs 1 and 2 for G and 2D to get the net frequency shift and solving them for ε and *n*(42,46)
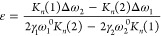
1
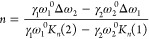
2Equations 1 and 2 are used for calculating the constant ε and *n* lines for Figure 3f, which are drawn for an increment of 0.1% strain and 5 × 10^12^ cm^–2^ doping, respectively. The zero-strain and zero-doping lines are the Raman frequencies of free-standing graphene.^42,47^Figure 3f compares the pristine graphene, heterostructure, and annealed graphene data. The pristine graphene is found to be p doped with 1 × 10^13^ cm^–2^ doping and almost ∼0% strain. On the other hand, the heterostructure shows a completely different behavior, revealing neutral graphene with a compression of −0.3%. It is worth noting that the compression value is much higher than the reported values typically observed in the wet-transferred and dry-transferred heterostructures, which generally range from about −0.1%. Figure S8 shows the example of the Lorentzian line shape fitting used to get the G and 2D peak positions. This observation affirms the epitaxial growth, where lattice mismatch introduces compression in graphene. The interaction between graphene and MoS_2_ within this heterostructure is more pronounced due to the direct growth method. This neutral behavior of graphene indicates a charge transfer of ∼10^13^ cm^–2^ from MoS_2_ to graphene. However, it is essential to note that this charge transfer behavior does not directly represent the ground state charge transfer. This discrepancy arises because the laser used for Raman measurements has a wavelength of 532 nm (∼2.3 eV), which exceeds the band gap of monolayer MoS_2_ (∼1.9 eV).^48^ Consequently, photoexcited electrons are generated in the conduction band of MoS_2_, leading to the observed charge transfer from MoS_2_ to graphene. To confirm this hypothesis, we grew the heterostructure on sapphire substrates. Subsequently, we studied the heterostructure using 532 (above the band gap) and 785 nm (below the band gap) wavelengths, as shown in Figure 3g. The 532 nm wavelength yielded a doping level similar to that measured over the Si/SiO_2_ substrate in Figure 3f, while the 785 nm wavelength showed a slight p-type doping effect. However, in the case of the 785 nm wavelength, the shift was not very distinct and fell within the error bar range. To better understand the ground state charge transfer behavior, we conducted transport measurements on the heterostructure and compared them with pristine graphene, as illustrated in Figure 4.

Figure 4a shows a multiterminal device nanofabricated via e-beam lithography patterning, metal evaporation, and lift-off. We conducted the four-probe gate-dependent measurements to gain insights into the electrical properties and the impact of MoS_2_ on graphene. In Figure 4b, a comparative analysis illustrates the gate-dependent drain-to-source voltage of bare graphene and the GRMS heterostructures with 55%, 72%, and 100% coverage by monolayer MoS_2_ on top. As seen here, MoS_2_ coverage of graphene in the heterostructure results in a noticeable shift toward positive gate voltage, indicative of p-type doping. The charge neutrality point (*V*_CNP_) is identified for bare CVD graphene at ∼81 V. In the case of the heterostructure, the devices exhibit a positively shifted *V*_CNP_ upon integration of MoS_2_ with *V*_CNP_ shifts of 10, 14, and 39 V for MoS_2_ coverages of 55%, 72%, and 100%, respectively, as exemplified in Figure 4c. These consistently show an electron transfer from graphene to MoS_2_ upon interfacial contact.^49−52^ Employing *n*_2D_ = *C*_ox_Δ*V*_CNP_/*e* (where *C*_ox_ is the parallel plate capacitance of the dielectric, Δ*V*_CNP_ is the change in the charge neutrality point, and *e* the electronic charge),^53,54^ the calculated charge transfer values are ∼7 × 10^11^, 1.0 × 10^11^, and 1.7 × 10^12^ cm^–2^ for MoS_2_ coverages of 55%, 72%, and 100%, respectively. Such a charge transfer could be attributed to an interlayer surface charge transfer between 2D materials and sp^3^ bond formation at the interface between graphene and MoS_2_. In addition to such natural carrier doping, photocarriers can be generated in MoS_2_, leading to enhanced conductivity in graphene. To study this, we performed photoconductivity measurements on our devices.

The depicted photoresponse of the device, showcased in Figure 4d, illustrates a characteristic negative photoresponse inherent to the graphene/MoS_2_ heterostructure. This behavior can be explained by CVD graphene’s typical p-type conductive nature, where the dominant charge carriers are holes. Upon laser irradiation, the photogenerated electrons within the MoS_2_ layer migrate to the graphene layer under the influence of an effective electric field resulting from the combined influence of an applied electric field, and a built-in interface electric field. Theoretically, the built-in electric field is sensitive to the p doping of graphene. In the case of the heterostructure, the presence of enhanced sp^3^ carbons can lead to highly doped graphene, resulting in a substantial built-in field that separates the electron holes in MoS_2_, consequently leading to a strong photoresponse. This migration of electrons from MoS_2_ to graphene causes a reduction in the majority carriers of graphene, i.e., holes, which elevates the Fermi level and consequently reduces conductivity, resulting in a negative photoresponse. The holes remain localized within the MoS_2_ layer due to extended charge-trapping times, confirming a charge transfer from MoS_2_ to graphene when exposed to light. In Figure 4e, the spectral response of the device displays a photoresponse in the range from 405 to 785 nm. The calculated photoresponsivity of the device is approximately 3 × 10^5^ mA/W with a time response of 1.2 s, as detailed in the Supporting Information (Figure S9), which is a promising value for directly grown CVD material heterostructure. These measurements are consistent with the earlier Raman spectroscopy measurements with low-wavelength excitations where n-type doping is observed. Thus, graphene gets p-type doped by MoS_2_ coverage, and photoconductance reveals that optically, n-type carriers can be induced into graphene from MoS_2_. First-principles density functional theory (DFT) calculations show slight p doping, as evident from the Fermi level shift (Figure S10c), which is in agreement with the experimental results. A detailed photocurrent map of the device demonstrates a direct correlation between enhanced photocurrent and the presence of monolayer MoS_2_ as displayed in the inset of Figure 4f. The defects present at the interfaces of graphene and MoS_2_ can serve as sites to capture and store charge carriers, which can impact photoluminescence (PL) and photoconductivity.^55^ However, considering GRMS heterostructures are largely van der Waals structures (as confirmed by the Raman spectrum), despite sparsely populated defects, the observation of PL and photoconductivity can be primarily attributed to the GRMS. This shows promise for designing graphene circuits with integrated optical sensors, photodetectors, and novel 2D optoelectronic and optospintronic sensors.

**Figure 5:**
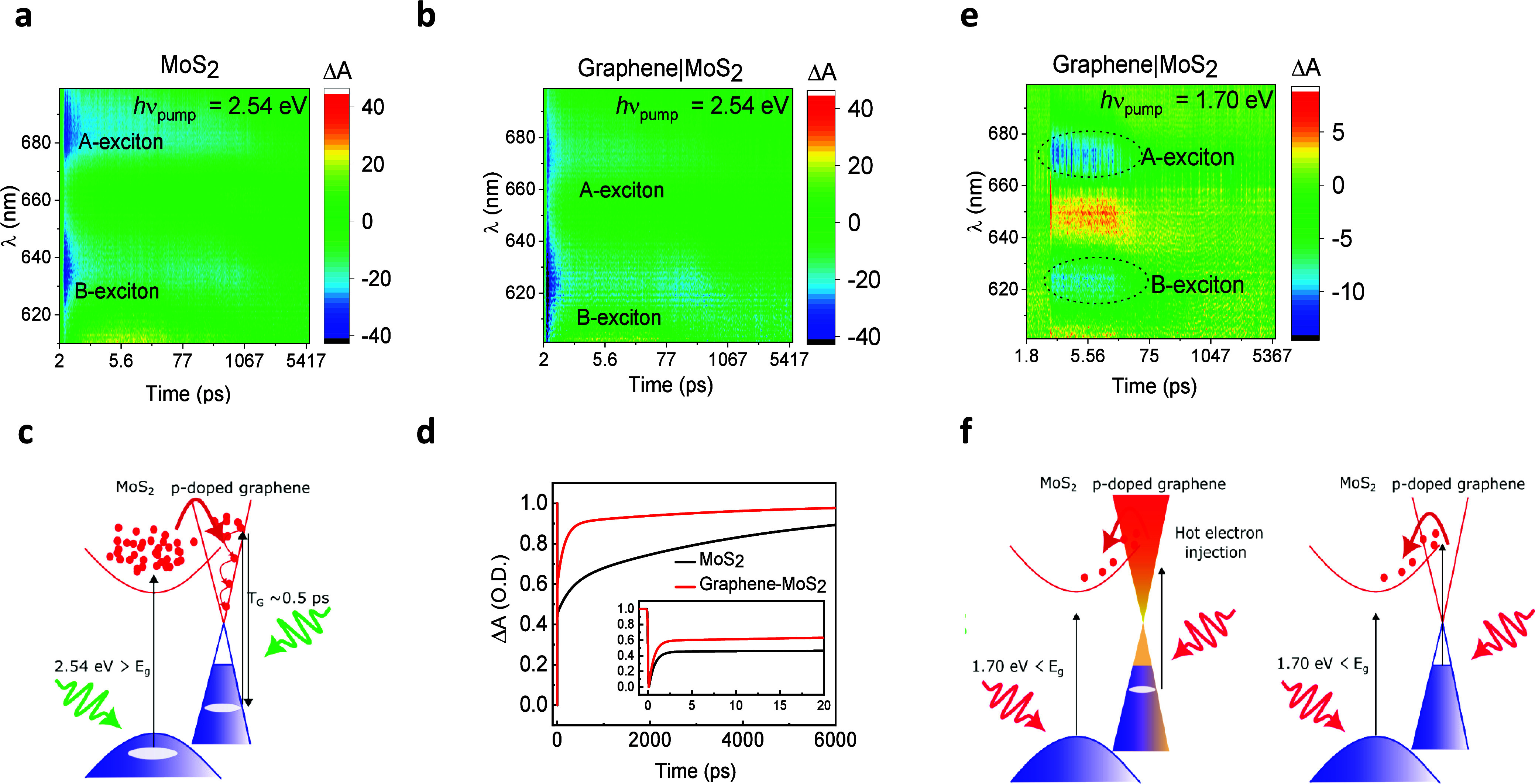
Pump–probe transient absorption (TA). Two-dimensional contour plots of the transient absorption for (a) monolayer MoS_2_ and (b) Graphene/MoS_2_ heterostructure, both synthesized using confined space chemical vapor deposit. The pump excitations had an average power of 0.5 mW and energy of 2.54 eV in a and b with the probing delays ranging from a few femtoseconds to 6 ns. (c) Possible charge transfer mechanism schematics for a pump energy (2.54 eV) higher than the band gap of MoS_2_ (1.9 eV). (d) Comparison of normalized transient dynamics of the MoS_2_ and GRMS heterostructure at a pump energy above the MoS_2_ band gap (2.54 eV). (e) Pump–probe TA 2D counter of the Graphene|MoS_2_ heterostructure utilizing a pump energy of 1.70 eV. This energy in e is intentionally below the MoS_2_ band gap, facilitating upconversion. (f) Possible charge transfer mechanism schematics: (left) pump energy (1.7 eV) is lower than the band gap of MoS_2_ (1.9 eV) and charge transfer involves hot electron injection from graphene to MoS_2_ due to electron excitation from the deep valence band of graphene, and (right) pump energy (1.7 eV) is lower than the band gap of MoS_2_ (1.9 eV), where charge transfer happens from graphene to MoS_2_ via the direct transfer method.

To understand the wavelength- and time-dependent ultrafast dynamics of GRMS heterostructures, we performed pump–probe transient absorption (TA) spectroscopy conducted under ambient conditions and at room temperature. TA spectroscopy allows uncovering the nonequilibrium dynamics of photocarriers within both heterostructure and pristine MoS_2_ samples. The experimental setup involves irradiating the materials with pump pulses above and below the MoS_2_ band gap energy (∼2.54 and 1.7 eV) and probing with white light. Detailed information regarding the experimental methodology is presented in the Experimental Section. MoS_2_ exhibits A and B excitonic absorption regions of MoS_2_, resulting from the splitting in the valence band of MoS_2_ due to high spin–orbit coupling. Figure 5a and 5b shows the TA two-dimensional contour plots for monolayer MoS_2_ and the heterostructure, respectively. Notably, the TA response captured in Figure 5a illustrates two distinctive features that specifically correspond to two ground state bleaching peaks, the A and B excitons at 685 and 637 nm, respectively.^56^ A positive feature is also observed, signifying broad photoinduced absorption under the pumping conditions. The reason behind the observed phenomena lies in the significant energy discrepancy between the pump pulse and the MoS_2_ band gap.^56,57^ Consequently, this energy input generates electrons (holes) at elevated (reduced) excitonic states within the conduction (valence) bands. These photoexcited electrons and holes subsequently undergo relaxation processes, forming B excitons, which further relax to A excitons at the lower valence band edge.^56^ This sequential relaxation accounts for the slower decay observed in the A exciton compared to the B exciton, as depicted in Figure 5a.

All samples are stimulated by a pump laser with an energy of 2.54 eV. However, the resultant spectra are predominantly governed by the A and B excitonic peaks of MoS_2_, as shown in Figure 5b. This dominance can be ascribed to the higher light-absorbing capacity of monolayer MoS_2_, approximately 10%, in contrast to monolayer graphene, which demonstrates only about 2% absorption.^58^ Notably, the A and B excitonic absorption peaks experience a shift to shorter wavelengths, specifically at 675 and 625 nm, respectively. This observed shift could result from band gap renormalization, corroborated by photoluminescence (PL) measurements in Figure S11 in the Supporting Information.^55^ As seen in Figure 5b, in the heterostructure, the amplitudes of both A and B excitons experience a notable reduction owing to a diminished population of excitons formed due to charge transfer to graphene.^57^ This observation validates the presence of efficient interfacial charge transfer from MoS_2_ to graphene, even before exciton formation. The kinetics of both MoS_2_ and the heterostructure are depicted in Figure 5d, with the GRMS kinetics exhibiting a markedly faster decay than pristine MoS_2_. The slower decay in pristine MoS_2_ is primarily attributed to the higher exciton formation, characterized by lifetimes in a few nanoseconds.^56,57,59^ The kinetics of both MoS_2_ and the GRMS heterostructure were analyzed employing three exponential components , where , *B*_*i*_, and *T*_*i*_ denote the normalized absorbance change, the fractional amplitude, and the decay constant, respectively (details for the fitting are given in Figures S12 and S13). Such analysis reveals more rapid dynamics in the heterostructure compared to pristine MoS_2_ and other previously reported TMDs and heterostructures as outlined in Table 1, thus affirming the efficiency of interlayer charge transfer.^55,57,59^ The fitted curves are provided in the Supporting Information, Figure S13.

**Table 1:** Fitting Parameters for Kinetic Curves of A and B Excitons and Global Fitting Parameters of Bare MoS_2_ and the Graphene-MoS_2_ Heterostructure in Figure 5a, 5b, and 5d

	MoS_2_ (pump = 2.64)			Graphene-MoS_2_ (pump = 2.64)			Graphene-MoS_2_ (pump = 1.70)		
samples	A	B	global	A	B	global	A	B	global
*t*_1_(ps)	0.64 (81.5%)	0.62 (74.8%)	0.64	0.57 (86.2%)	0.57 (81.5%)	0.57	0.465 (66.9%)	0.266 (46.1%)	0.32
*t*_2_(ps)	297.7 (13.2%)	300.3 (22.7%)	297.3	147.2 (9.07%)	152.1 (13.9%)	148.50	34.2 (20.3%)	47.5 (50.3%)	12.24
*t*_3_(ps)	4.62E3 (5.2%)	4.61E3 (2.37%)	4.61E3	4.06E3 (4.85%)	4.07E3 (4.54%)	4.05E3	4.7E3 (12.7%)	50.5E3 (3.52%)	4.5E3

In order to explore the upconversion in heterostructures, we explored the heterostructures when illuminated with a 1.7 eV pump laser, notably below the band gap of monolayer MoS_2_ (1.8 eV). In this scenario, only graphene is photoexcited within the heterostructure. Strikingly, even in this case, the distinct ground state bleaching of the A and B excitons of MoS_2_ remains distinctly observable (Figure 5e), which is attributed to a reverse charge transfer process from graphene to MoS_2_. The reverse charge transfer process can be explained through direct transfer (DT) and/or hot carrier transfer (HET), as depicted in Figure 5c (DT) and 5f (HET and DT), with band alignment showing that the Dirac point of graphene resides within the band gap of MoS_2_.^59^ The direction of charge transfer is found to be dependent on the pump energy. When the pump energy (2.54 eV) exceeds the band gap of MoS_2_, both MoS_2_ and graphene exhibit photoexcited carriers.^60^ The photocarriers in graphene are reported to rapidly decay (approximately 0.5 ps) compared to TMDs with slow decay kinetics due to the formation of excitons.^60^ This results in the transfer of electrons from MoS_2_ to graphene (as shown in Figure 5c), thereby justifying the faster kinetics and reduced PL in GRMS. On the other hand, when the GRMS is irradiated with a 1.7 eV laser, electrons are excited in graphene solely. This photoexcited electron can transfer from graphene to MoS_2_, causing ground state bleaching of A and B excitons in this scenario (see Figure 5e). The first possible mechanism is HET, as depicted in Figure 5f, which has been documented in similar systems like WS_2_/graphene.^55,57^ Photoexcited electrons and holes with energy *E* = *E*_photon_/2 establish a quasi-equilibrium distribution within graphene with an elevated effective electron temperature (*T*_e_) due to carrier–carrier scattering.^61^ The photoexcited electrons must possess an energy higher than the Schottky barrier (φ) between graphene and MoS_2_, which is equivalent to the difference between the conduction band minimum of MoS_2_ (*E*_CBM_) and the Dirac point of graphene (*E*_D_). At *E*_photon_ = 1.7 eV, the number of electrons with this requisite energy follows a Boltzmann distribution, scaling as exp (−φ/*K*_B_*T*), where *K*_B_ represents the Boltzmann constant and *T* is the temperature.^61^ Another feasible method is a direct transfer (DT), as shown in Figure 5f (left), when the electrons are excited from the Fermi level of graphene possessing sufficient high energy.^55^ We conducted fluence-dependent absorption measurements in GRMS for both pumps above and below the MoS_2_ band gap, as shown in Figure S14. Direct absorption typically displays linear fluence dependence (*P^a^*, *a* = 1), while hot carrier injection shows superlinear fluence dependence with *a* > 1.^57,61^ In our case, the exponent *a* is approximately 0.95 for *E*_pump_ = 2.5 eV, while it increases to 1.35 for *E*_pump_ = 1.7 eV, clearly indicating a substantial contribution from hot carrier injection from graphene to MoS_2_, resulting in the upconversion of electrons to higher energy levels in MoS_2_.

## Conclusion

In summary, we have demonstrated the direct growth of efficient MoS_2_ monolayers on patterned graphene through a confined space chemical vapor deposition growth technique. Extensive characterizations using wavelength-dependent Raman spectroscopy, electrical measurements, and photoconductivity confirm the p-type doping in graphene due to the top layer of MoS_2_. Raman measurements revealed an electron transfer of ∼10^13^ cm^–2^ from MoS_2_ to graphene, accompanied by a 0.3% compressive lattice strain in graphene. Photoconductivity measurement shows that photoactive regions can be created locally on graphene channels via local MoS_2_. Time-resolved ultrafast transient absorption spectroscopy unveils controlled charge transfer kinetics in GRMS heterostructures with accelerated photocarrier decay due to the presence of graphene. Furthermore, it reveals an upconversion within the GRMS, as evidenced by observing ground state bleaching corresponding to the A and B excitons of MoS_2,_ even under below band gap excitation conditions. Our results pave the way for the direct growth of MoS_2_ monolayers and other transition metal dichalcogenides (TMDs) on patterned graphene channels for ultrafast photoactive nanoelectronics and spintronic circuits.

## Experimental Section

### Growth of MoS_2_ on Patterned Graphene

Commercial CVD graphene from Graphenea on Si/SiO_2_ was patterned using electron beam lithography (EBL) with negative tone photoresist ma-N 2403 coated on graphene and developed using ma-D 525. The rest was etched away by oxygen plasma with 50 W for 45 s. The resist was removed using a lift-off procedure with 70 °C acetone and rinsing with room-temperature acetone and IPA. Patterned graphene stripes had a length of 200 μm, varied widths of 30–10 μm, and a constant spacing of 40 μm. The graphene stripes on a Si/SiO_2_ substrate were introduced into a three-zone furnace, where an Ar/H_2_ inert atmosphere was established for the subsequent growth of MoS_2_. For this growth process, MoO_3_ powder (Sigma-Aldrich, 99.995%) and sulfur powder (Sigma-Aldrich, 99.995%) were used as precursor materials. A continuous gas flow of 650 standard cubic centimeters per minute (sccm) was maintained throughout the growth process. The growth temperature was set at 740 °C and held constant for 4 min. Subsequently, the furnace was abruptly opened to terminate the growth process, forming monolayer MoS_2_ on the graphene stripes. Once the growth was arrested and cooled to room temperature, the GRMS heterostructures were carefully removed from the furnace.

### X-ray Photoelectron Spectroscopy (XPS)

The electronic structure of the Graphene/MoS_2_heterostructure was investigated using XPS and compared to pristine graphene and pristine MoS_2_. Core levels and overview spectra were obtained using an excitation energy of 1486.6 eV (Al Kα) with an energy resolution of 0.25 eV. Hard X-ray photoelectron spectroscopy was utilized to measure the MoS_2_ S 1s core level, employing a Ga Kα source with an excitation energy of 9252.8 eV and an energy resolution of 0.5 eV. The step size for core-level spectra was 0.1 eV, while for overview spectra, it was 0.5 eV. A pass energy of 200 eV was used for all measurements. The Igor Pro 7.08 software and a Curve Fitting procedure were employed to analyze the core-level spectra. Voigt functions were utilized to fit the core level peaks, while the inelastic background was modeled using a Shirley function.

### Device Fabrication

The MoS_2_ monolayers grown over graphene were identified, and devices were designed. Using EBL and a digital mask designed specifically for the Graphene/MoS_2_ heterostructure, we patterned the devices using a positive tone photoresist. We used a double-layer resist of MMA EL 9 and ARP 6200.13. After the exposure in EBL, the development was done in hexyl acetate, MIBK (methyl isobutyl ketone)/IPA, and IPA. The 5 nm Ti and 55 nm Au layers were deposited using an e-beam evaporator for the electrodes and contact pad. After lift-off with hot acetone and rinsing with room-temperature acetone and IPA, the device was realized.

### Auger Measurements

Auger measurements were carried out on an Auger microprobe Jeol JAMP-9510F with a Schottky electron emitter and hemispherical electron analyzer using an excitation primary beam with 10 keV energy and 11 nA current. The beam incidence angle was 30° with an emission angle of 25°. Auger spectra were recorded in direct form in a constant retarding ratio regime with energy resolution of 0.5% at 100 ms dwell time and 1 eV step and are presented here in derivative form.

### Raman Spectroscopy

Raman spectra and Raman mapping were collected using Renishaw Qontor and Renishaw InVia spectrometers. The samples were examined by using a frequency-doubled Nd:YAG laser to obtain 532 nm and a solid state laser for 785 nm, with the Rayleigh line removed by edge filters of optical density > 6 and cutting 85 and 35 cm^–1^ into the Stokes side, respectively. Gratings and slits were utilized to ensure a Raman resolution of less than 1 cm^–1^ per pixel.

### Electrical Measurements

Electrical characterization of the contacts and the device was performed using multiterminal measurement geometry at room temperature. Electrical transport measurements were performed in high-vacuum conditions (∼10^–7^ mbar) using a Keithley current source and a nanovoltmeter. In addition, a Keithley source meter applied gate voltage.

### Transient Absorption Spectroscopy and Analysis

An 800 nm pulse with a 120 fs duration is generated by a Ti:Sapphire (Spectra-Physics, 1 kHz, 5 mJ) laser system. A segment of the beam (1.68 mJ) is employed to generate the resonant pump pulse through an optical parametric amplifier for exciting electronic transitions in the photosystem. A mechanical chopper, synchronized with the amplifier, blocks alternate excitation pulses. Another beam segment (0.78 mJ) passing through a CaF_2_ window creates a white light continuum used as a broad-spectrum probe. It is important to highlight that we have only focused on excitonic peaks, thus measuring the transient spectra in the 600–700 nm regime. Spatial and temporal overlap of the probe and pump beams occur within the sample. Postsample, the reflective probe beam is detected after blocking the pump beam. An optical delay stage (0–6 ns) varies the time delay between the pump and the probe pulses. The pump pulse power (0.5 mW) remains low to prevent sample degradation and multiexciton effects. The instrument response function is around ∼170 fs at the sample position. Extracting TA data involves Glotaran-assisted global analysis. Extracting TA data involves Glotaran-assisted global analysis. *T*_*i*_, representing decay constant, can be explained corresponding to different phenomena for *i* = 1–3.^62−64^*T*_1_ (∼1–5 ps) is assigned to defect-assisted carrier cooling, *T*_2_ (10–100 ps) to exciton–phonon scattering, and *T*_3_ (a few hundred picoseconds) to the recombination of electron and hole pairs of excitons responsible for photoluminescence (PL). Additionally, the possibility of a fourth time constant *T*_4_ (approximately tens of picoseconds) exists assigned to exciton–exciton interactions, leading to annihilation or biexciton formation.^62^ We chose three exponential components based on the observation that charge transfer decreases exciton population density, reducing the likelihood of biexciton formation or exciton–exciton annihilation (EEA). We compared the time constants and found a significant decrease in the first two components (*T*_1_ and *T*_2_) due to charge transfer from MoS_2_ to graphene, providing a faster decay channel.

### HAADF-STEM Measurements

An electron transparent lamella was prepared using a Scios 2 DualBeam (Thermo Fisher Scientific) FIB-SEM system (Ga+ FIB) with a target size of 12 × 5 μm, thickness < 50 nm, and deposition of a protective Pt layer. A thin amorphous carbon layer around 10 nm thick was deposited using arc discharge deposition. The final thinning process consisted of 30 kV thinning, 5 kV, and 2 kV polishing. HAADF-STEM imaging of the lamella was carried out using an aberration-corrected scanning transmission electron microscope, Jeol JEM-ARM200cF, operated at an acceleration voltage of 200 kV. The convergence semiangle of the incident beam probe was set to 15 mrad. STEM images were collected using a HAADF detector having inner and outer semiangles of 45 and 180 mrad, respectively, and an annular bright-field (ABF) detector having inner and outer semiangles of 11 mrad. EELS elemental analysis was carried out using a Gatan GIF 965 Quantum ER camera equipped with a fast-Dual EELS system. EELS collection semiangle was set to 29.3 mrad. The energy dispersion was set at 0.25 eV/channel. All acquired data were processed with 64-bit Digital Micrograph GMS 3.21 (Gatan).

## Supporting Information Available

Additional experimental data through optical, elemental composition, and spectroscopy characterizations of GRMS and first-principles DFT calculation of the heterostructure (PDF)
