# Supplementary material for: Large-Scale Direct Growth of Monolayer MoS2 on Patterned Graphene for van der Waals Ultrafast Photoactive Circuits
Source: ACS Appl Mater Interfaces. 2024 Jul 12;16(29):38711–22. doi: 10.1021/acsami.4c07028 (PMC11284756; doi:10.1021/acsami.4c07028)
Supplement: Supplementary file 1 — am4c07028_si_001.pdf [file am4c07028_si_001.pdf]

Supplementary Information

Large-scale direct growth of monolayer MoS_2 on patterned Graphene for van der Waals ultrafast-photoactive circuits

Rahul Sharma[1†], Henry Nameirakpam[1†], David Muradas Belinchón[1], Prince Sharma[2], Ulrich Noumbe[1,6], Daria Belotcerkovtceva[1], Elin Berggren[1], Viliam Vretenár[3], Ľubomír Vančo[3], Matúš Maťko[3], Ravi K. Biroju[3, 4], Soumitra Satapathi[2], Tomas Edvinsson[5], Andreas Lindblad[1] and M. Venkata Kamalakar[1]*

[1]Department of Physics and Astronomy, Uppsala University, Box 516, SE-751 20 Uppsala, Sweden
[2]Department of Physics, Indian Institute of Technology Roorkee, Roorkee 247667, India
[3]Centre for Nanodiagnostics of Materials, Faculty of Materials Science and Technology, Slovak University of Technology, Vazovova 5, Bratislava 812 43, Slovakia
[4]School of Advanced Sciences−Division of Physics, Vellore Institute of Technology, Vandalur−Kelambakkam Road Chennai, Chennai, Tamil Nadu 600127, India
[5]Department of Materials Science and Engineering, Uppsala University, Box 35, SE-751 03 Uppsala, Sweden
[6]Université de Strasbourg, CNRS, Institut de Physique et Chimie des Matériaux de Strasbourg (IPCMS), UMR 7504, 23 rue du Loess, Strasbourg 67034, France

†co-first authors
*email to: venkata.mutta@physics.uu.se

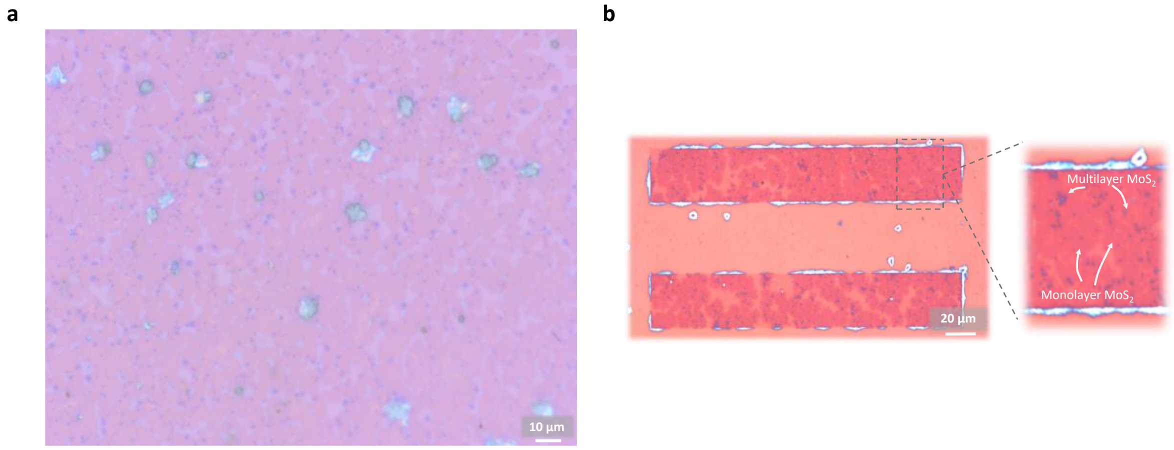

Figure S1. a) Large area monolayer growth of MoS_2 over graphene sheet, b) Optical images of MoS_2 almost covering the patterned graphene.

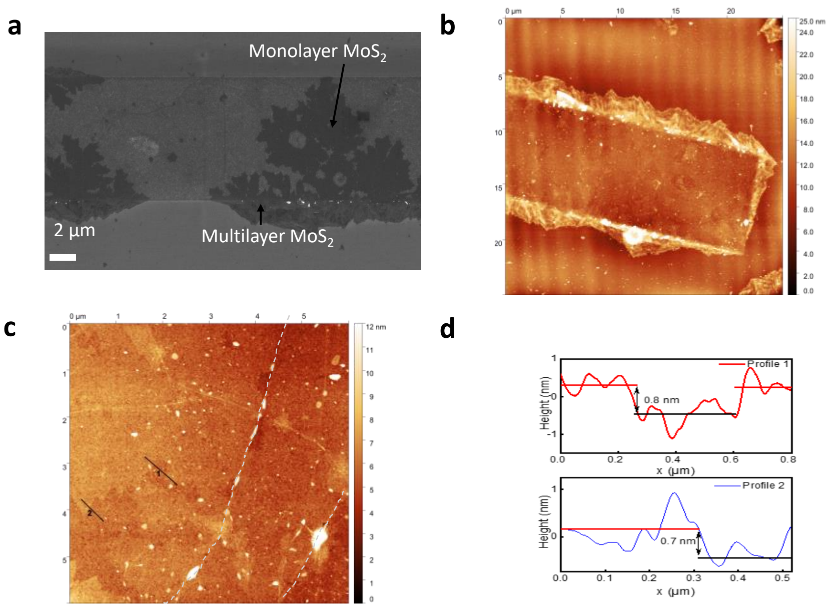

Figure S2. a) Images from field emission scanning electron microscopy (FESEM) showing a graphene strip covered with monolayer of MoS_2 and multilayer growth at the step edge. Atomic force microscopy images (AFM) of b) MoS_2 growth on graphene, c) monolayer of MoS_2 grown over the graphene strip. Grey dotted lines show grain boundaries on graphene. d) Line profiles derived from image c, illustrate the monolayer growth of MoS_2.

Table S1. Quantification of the elemental composition from the EELS spectrum.

Samples	E_{2g} peak	A_{1g} peak	$E_{2g}: A_{1g}$	G band	2D band
MS	385.1	403.7	0.75		
GR	-	-		1596.7	2682.6
GRMS	382.4	405.2	0.82	1587.3	2695.5
Change (Shift)	-2.7	1.5	-0.2	-9.4	12.9

E_{2g} shift = strain in MS (blue shift compression, red shift tensile strain)

A_{1g} = charge transfer in MS (blue shift p doping, red shift n doping)

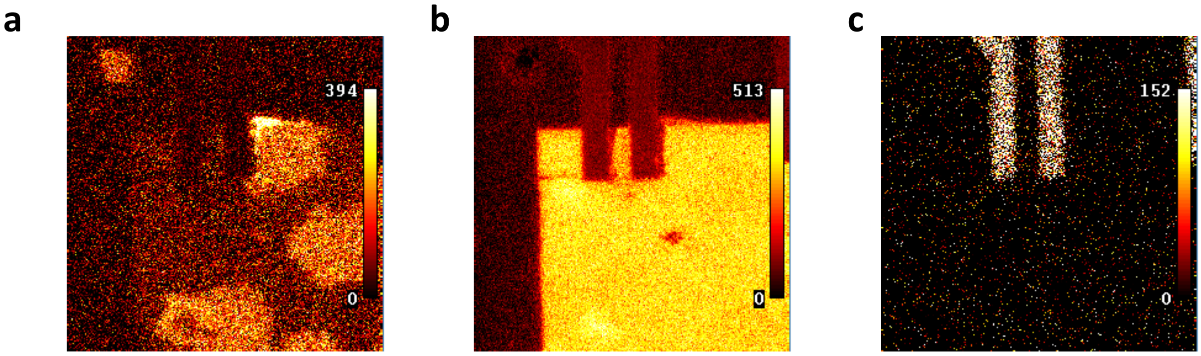

Figure S3. Auger mapping of the GRMS heterostructure illustrates contrast for monolayer graphene, MoS_2, and gold electrodes at a) S-LVV, b) C-KLL, and c) Au-MNN transitions.

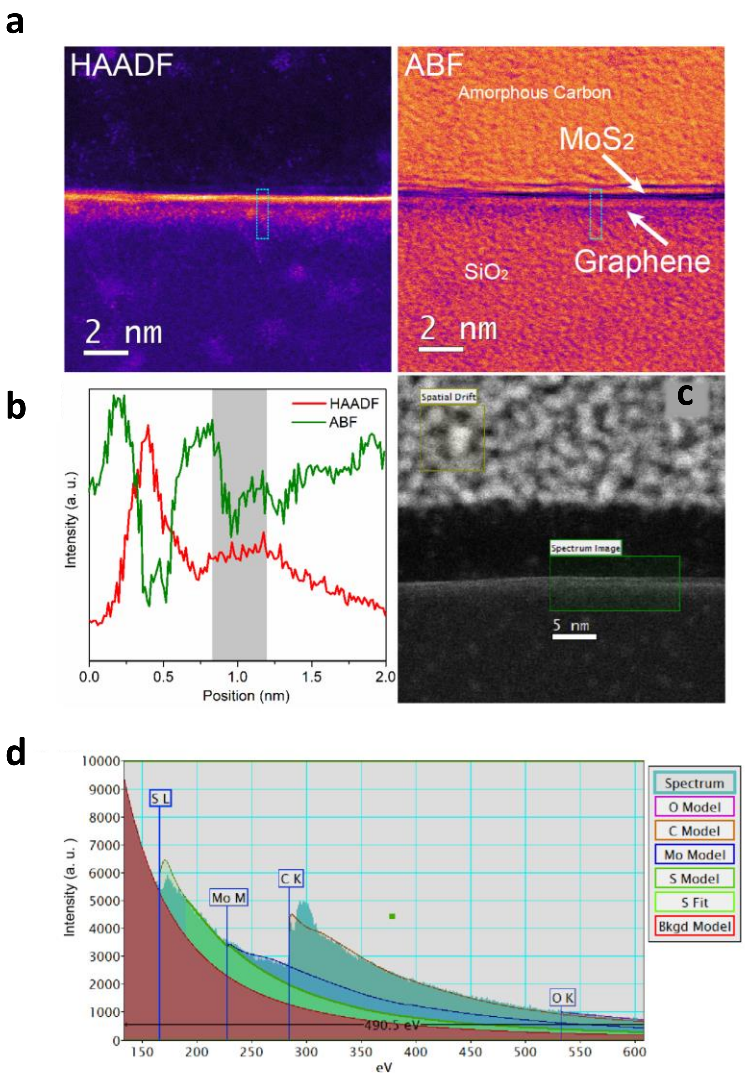

Figure S4. a) Cross-sectional HAADF and BF -STEM images of GRMS. b) The corresponding line profile is extracted from the marked region as shown in the HAADF and BF images at the same location. c) ADF image of a GRMS interface and corresponding survey image used for an EELS map. d) EELS spectrum at the vdW interface.

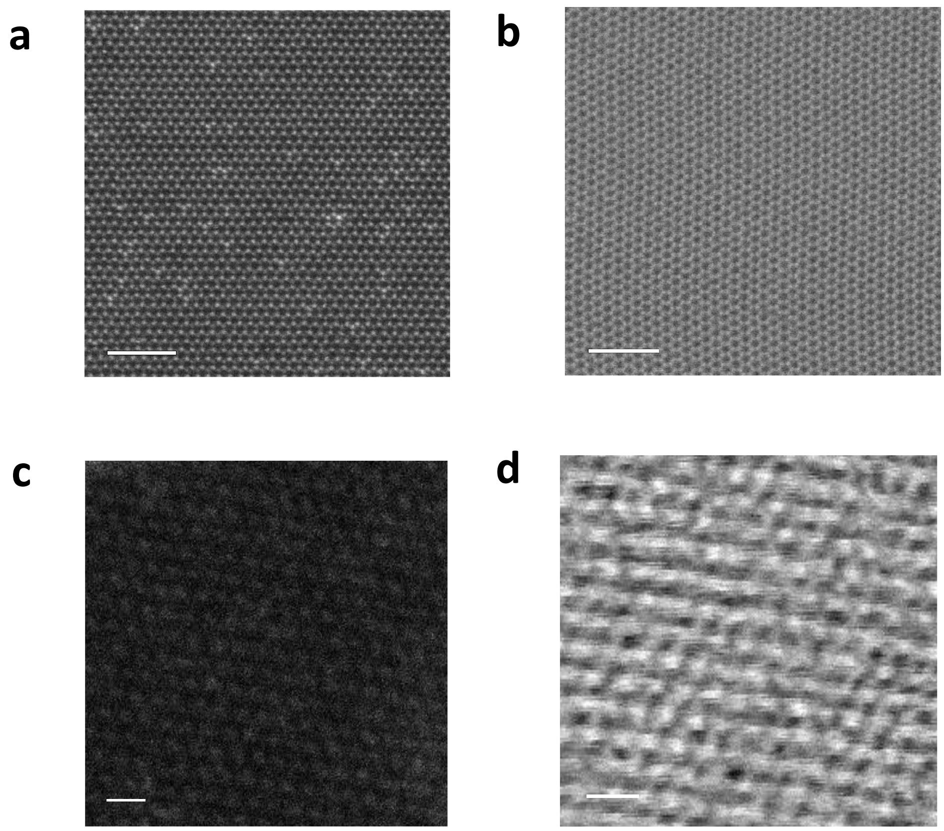

Figure S5. HAADF images of GRMS a) unprocessed, b) bright Field. HAADF images of monolayer graphene c) Unprocessed d) Bright Field.

Table S2. Quantification of the elemental composition from the EELS spectrum.

Sl. No	Element	Shell	Composition (at. %)
1	S	L	6.2±0.3
2	Mo	M	3.12±0.14
3	C	K	86±4
4	O	K	5.0±0.2

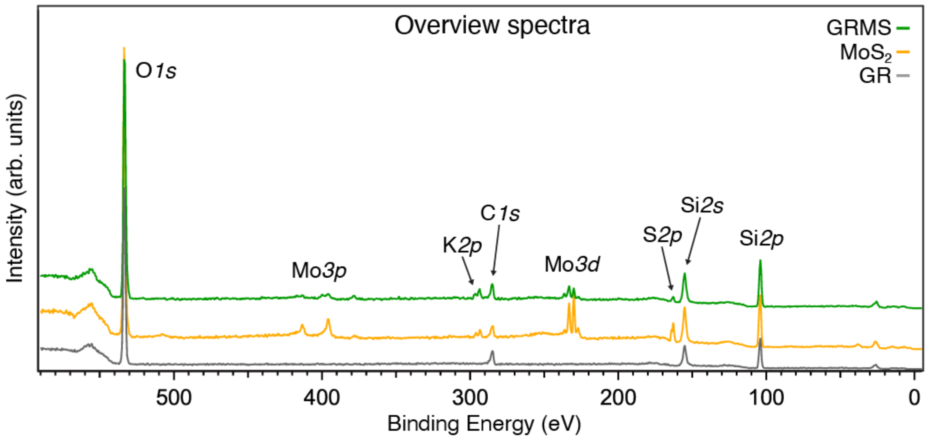

Figure S6. Survey spectra for Graphene, MoS_2, and GRMS.

Table S3. Photoelectron line positions and assigned compound for pristine MoS_2, graphene, and GRMS.

Graphene			MoS_2		**GRMS**	
Mo3d			BE (eV)	Area Ratio	BE (eV)	Area Ratio
Mo^{4+}			230.4 / 233.5	0.79	230.1 / 232.2	0.53
Mo^{6+}			233.6 / 236.7	0.21	233.6 / 236.7	0.47
C1s	BE (eV)	Ratio (area)			BE (eV)	Ratio (area)
C – C (sp^2)	284.8	0.49			284.8	0.41
C – C (sp^3)	245.7	0.24			245.7	0.34
C – OH	286.7	0.14			286.7	0.13
C – O	287.6	0.07			287.6	0.06
C = O	289.3	0.06			289.3	0,06
S2p			BE (eV)		BE (eV)	
S *2p*			162.9 / 164.1		162.6 / 163.8	
S – O			166.3		166.1	

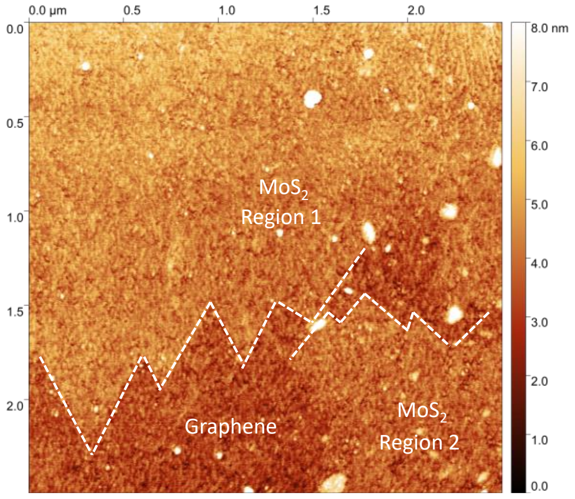

Figure S7. High-resolution AFM image of GRMS sample in single graphene crystal. The white dotted line is the guide for eyes to show the growth of MoS_2 crystals showing the same orientation for all crystals.

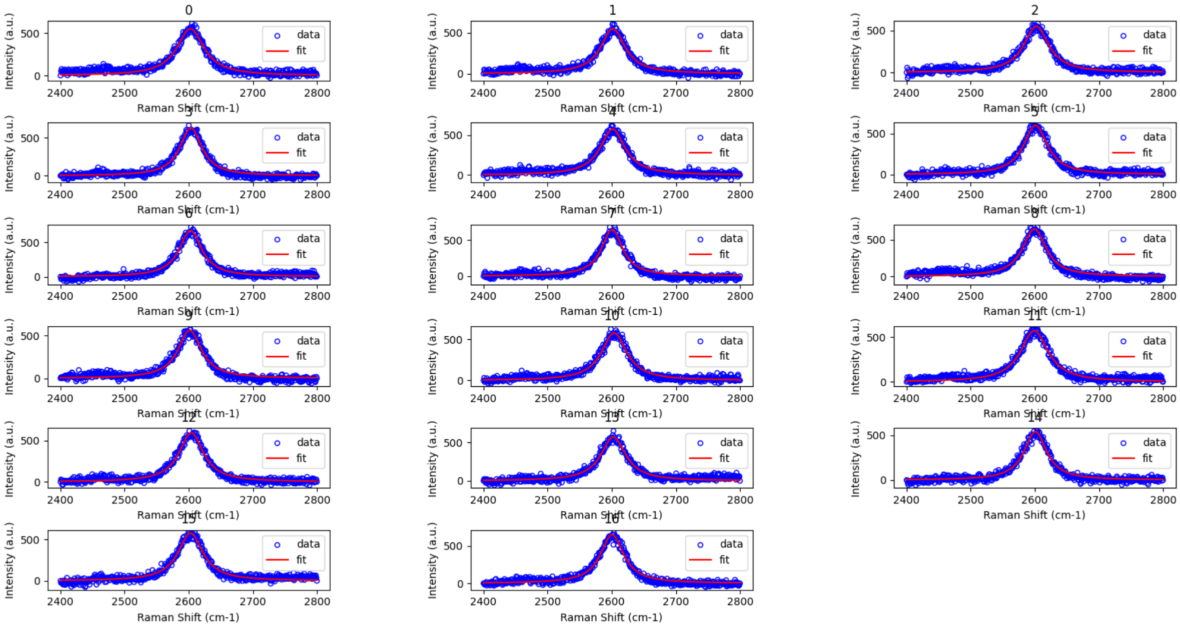

Figure S8. Example of Lorentzian Line Shapes fitting done at each point of the map for recreating maps from Raman Spectroscopy data.

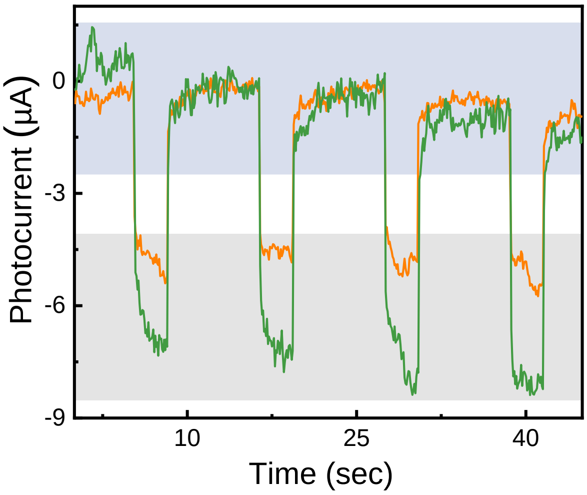

Figure S9. Time response of GRMS device for 532 nm (green) and 633 nm (orange) lasers.

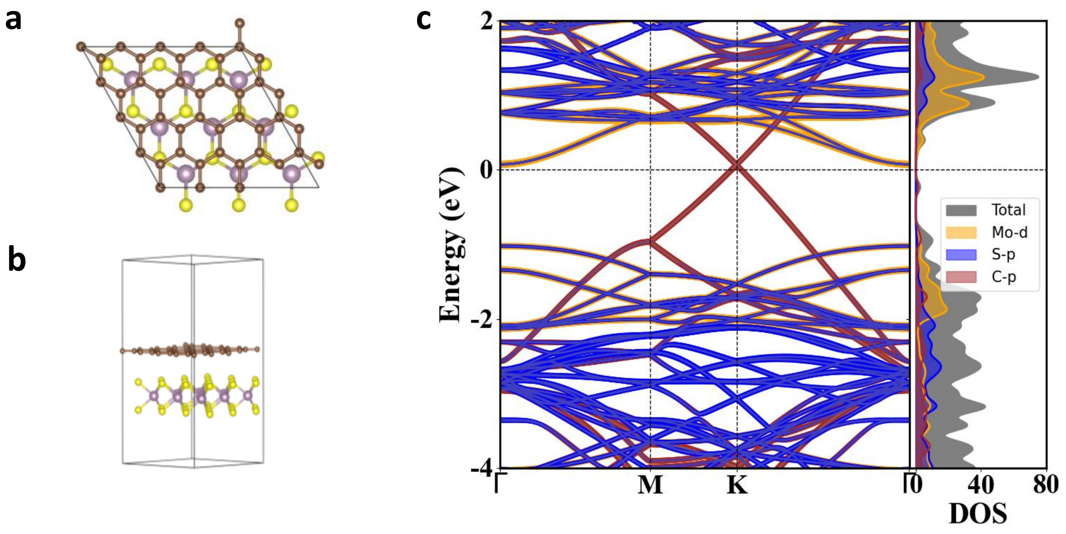

Figure S10. Schematic of graphene/MoS_2 heterostructure a and b showing top and side view respectively. c) Band structure and density of states (DOS) from the heterostructure.

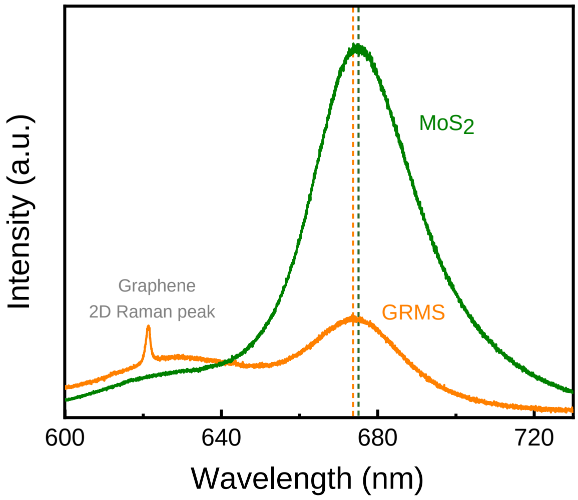

Figure S11. Photoluminescence comparison of MoS_2 and GRMS heterostructure.

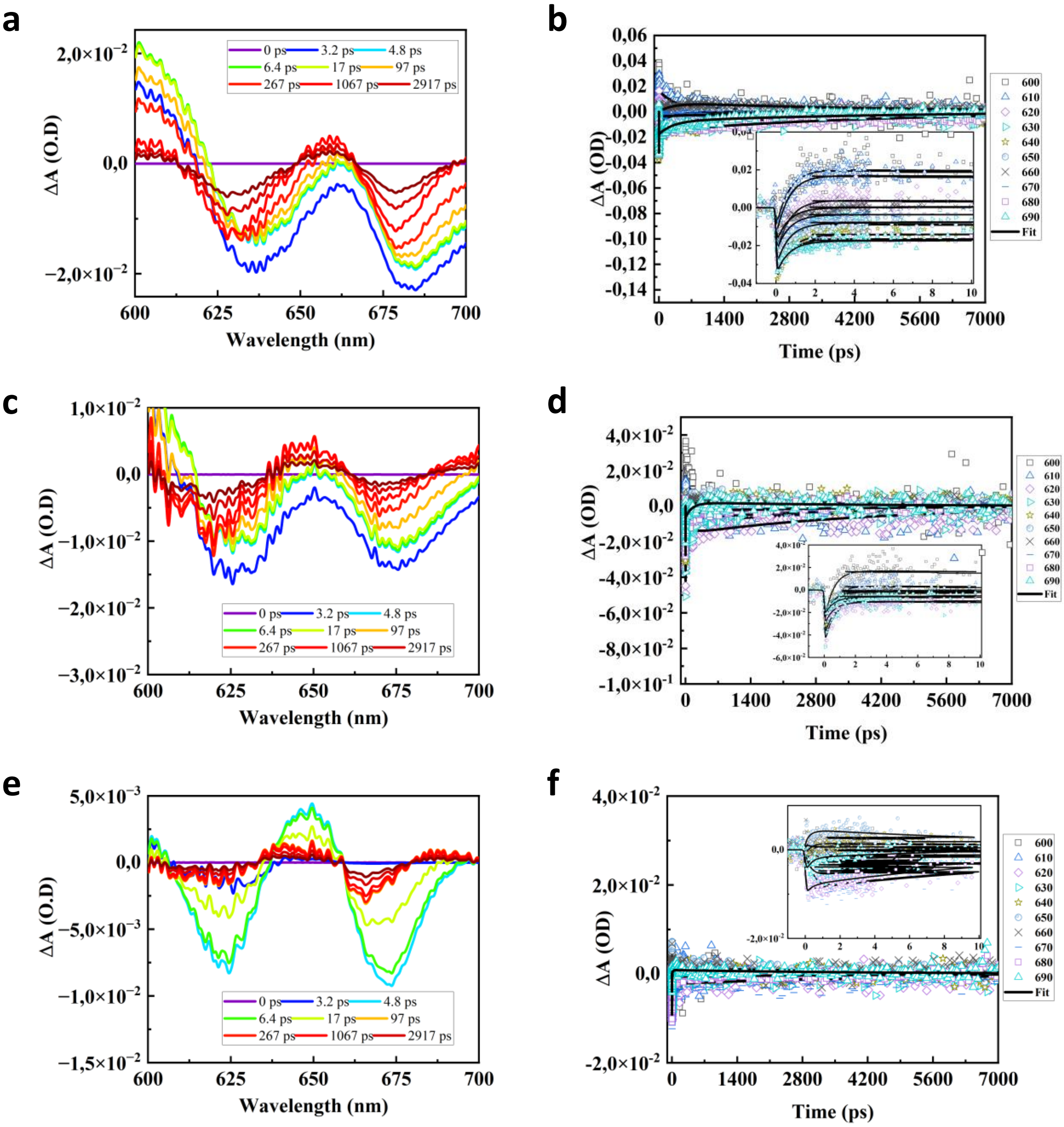

Figure S12. Global spectra and kinetic fitting through glotaran of MoS_2 (a and b), GMRS at a pump of 2.54 eV (c and d) and 1.70 eV (e and f).

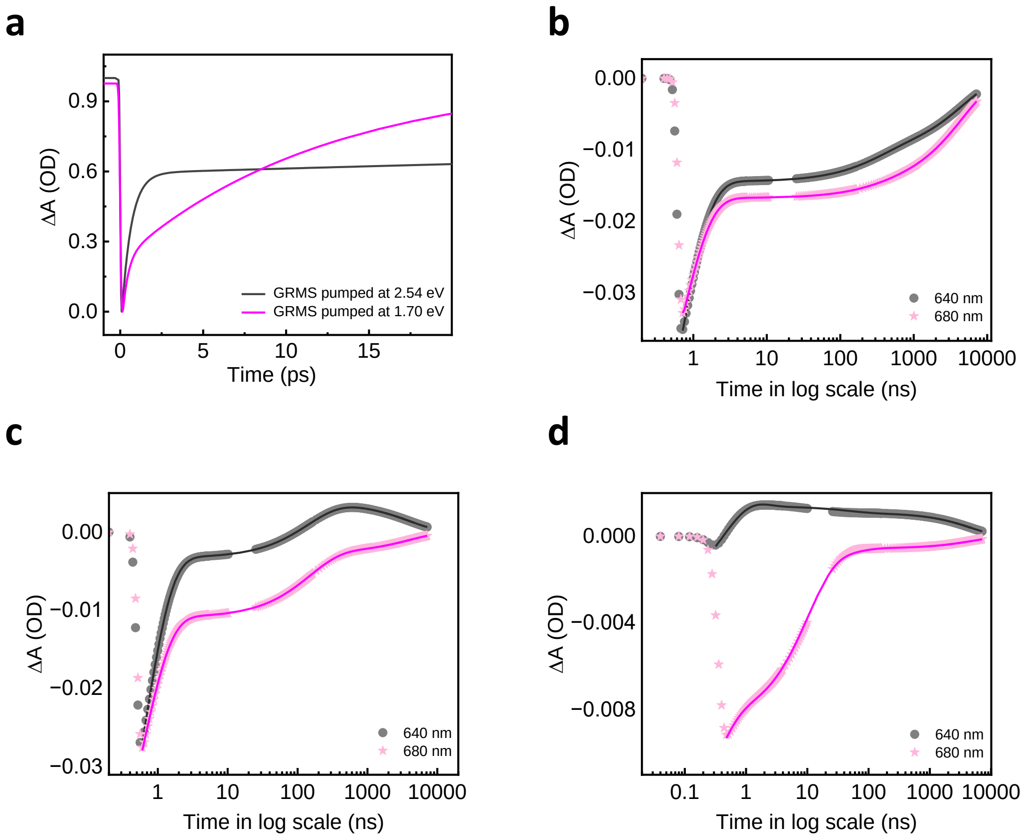

Figure S13. a) Normalised kinetic profile comparison of GRMS at a pump laser of 2.54 and 1.70 eV. Targeted kinetic fitting by considering three exponential decay lifetimes ($Y=A_1*\exp(-x/t_1) + A_2*\exp(-x/t_2) + A_3*\exp(-x/t_3)$) of b) MoS_2, c) 2.54 eV and d) 1.70 eV.

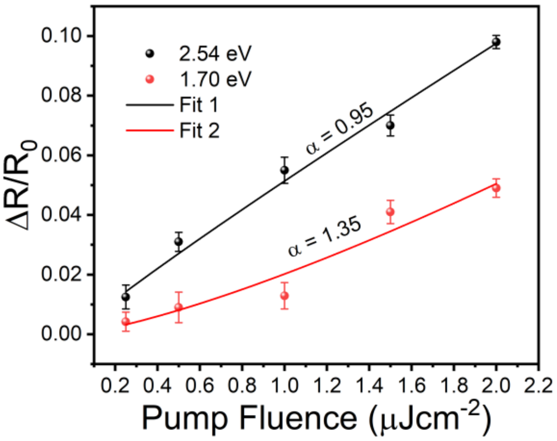

Figure S14. The amplitude of the TA signal at a 0-ps time delay versus pump fluence for GRMS heterostructure with pump energy above (black dots) and below (red dots) the optical gap. The continuous lines are the fit to the data with a power-law function.
